# Virtual embodiment for improving range of motion in patients with movement-related shoulder pain: an experimental study

**DOI:** 10.1186/s13018-023-04158-w

**Published:** 2023-09-26

**Authors:** Mercè Álvarez de la Campa Crespo, Tony Donegan, Beñat Amestoy-Alonso, Andrea Just, Andrés Combalía, Maria V. Sanchez-Vives

**Affiliations:** 1 Institut d’Investigacions Biomèdiques August Pi i Sunyer (IDIBAPS), Rosellón, 149, 08036 Barcelona, Spain; 2 Servicio de Rehabilitación y Fisioterapia, Quironsalud Barcelona, Plaça d’Alfonso Comín, 5, 08023 Barcelona, Spain; 3 Fundación Garcia Cugat, Quironsalud Barcelona, Plaça d’Alfonso Comín, 5, 08023 Barcelona, Spain; 4 Departament de Cirurgia i Especialitats Medicoquirúrgiques, Facultat de Medicina i Ciències de la Salut, Universitat de Barcelona, Casanova, 143, 08036 Barcelona, Spain; 5 Servei de Cirurgia Ortopèdica i Traumatologia, Hospital Clínic de Barcelona, Universitat de Barcelona, Villarroel, 170, 08036 Barcelona, Spain; 6 Facultat de Medicina i Ciències de la Salut, Universitat de Barcelona, Casanova, 143, Barcelona, Spain; 7 Institució Catalana de Recerca i Estudis Avançats (ICREA), Passeig de Lluís Companys, 23, 08010 Barcelona, Spain

**Keywords:** Virtual reality, Embodiment, Pain, Musculoskeletal, Rehabilitation, Body functionality

## Abstract

**Background:**

Recent evidence supports the use of immersive virtual reality (VR) as a means of delivering bodily illusions that may have therapeutic potential for the treatment of musculoskeletal conditions. We wanted to investigate whether a single session of an embodiment-based immersive VR training program influences pain-free range of motion in patients with shoulder pain.

**Methods:**

We designed a rehabilitation program based on developing ownership over a virtual body and then “exercising” the upper limb in immersive VR, while the real arm remains static. We then carried out a single-arm pre-post experiment in which 21 patients with movement-related musculoskeletal shoulder pain were exposed to the 15-min VR program and measured their active pain-free range of motion immediately before and afterwards.

**Results:**

We found that shoulder abduction and hand-behind-back movements, but not shoulder flexion, were significantly and clinically improved post-intervention and that the level of improvement correlated with the level of embodiment. Following this one session, at 1-week follow-up the improvements were not maintained.

**Conclusions:**

Virtual embodiment may be a useful therapeutic tool to help improve range of motion in patients with movement-related shoulder pain in the short term, which in turn could expedite rehabilitation and recovery in these conditions.

## Introduction

Musculoskeletal-related shoulder pain is a common and often highly disabling complaint with a lifetime prevalence of up to 70% [1]. The majority of cases of shoulder pain are thought to be related to the rotator cuff and include conditions such as subacromial bursitis, calcific tendinosis, supraspinatus tendinopathy, rotator cuff tendon tears, bicipital tendinitis or rotator cuff degeneration [2]; another common factor is adhesive capsulitis, in which the shoulder capsule becomes thickened and fibrosed, with severe limitation of movement and high pain levels, which is particularly frequent among patients with diabetes [3].

Recovery from these conditions is often slow. Twenty-five per cent of those affected by rotator-cuff-related shoulder pain (RCRSP) report previous episodes, and up to 50% of these patients still report persistent pain 6–12 months after initially seeking treatment [4]. In the case of adhesive capsulitis, recovery is very gradual, typically taking a minimum of 18 months, with pain easing first followed by gradual restoration of range of motion [3].

There are various interventions for these conditions, including physiotherapy (encompassing education, exercise, manual therapy, electrotherapy, etc.), corticosteroid injections, medication and surgery. However, conservative therapy is the mainstay of treatment, as clinical trials suggest that long-term results of patients treated surgically are comparable to those receiving rehabilitation [5]. Unfortunately, treatment is considered ineffective in more than one-third of the patients with RCRSP, who continue to have pain and disability post-treatment [6], and for adhesive capsulitis, physiotherapy combined with intra-articular steroid injection tends to provide only short-term pain relief and improved function for 4–6 weeks [3]. This apparent failure of conservative treatment in a significant proportion of cases, together with the fact that shoulder pain is often poorly related to pathological findings in the shoulder tissues [2], suggests a role for central nervous system changes, including sensitization at the spinal cord [7] and maladaptive cortical changes [8, 9]. Indeed, signs and symptoms of central sensitization have frequently been found in people with shoulder pain [10, 11].

A movement or activity that is repeatedly experienced as painful over prolonged periods may eventually be enough to trigger a painful experience even with comparatively reduced nociceptive input (when peripheral sensitization has eased); for example, via the sensitization of second-order nociceptive neurons [12], which is thought to occur through a combination of reduced firing thresholds and/or reduced cortical inhibition. Normal and healthy physiological movements and activities—which would ordinarily be therapeutic—may therefore be experienced as painful above a certain range. In both acute and chronic shoulder pain, movements below around 60° of shoulder abduction and flexion are typically relatively pain-free but become painful above these ranges [13]. Hand-behind-back movements (involving shoulder internal rotation and extension) are often also restricted and painful. In acute conditions, the range of motion at which pain is felt gradually increases as the patient recovers, while in persistent pain these range-of-motion losses often persist (even when the pathology appears to resolve, or the suspected painful tissue is excised or otherwise treated surgically). Measuring pain-free range of motion is therefore a useful outcome measure for assessing overall progress.

The degree of central sensitization may be associated with cognitive factors such as kinesiophobia or pain catastrophizing [6], as well as psychological factors such as stress, anxiety or depression [14]. Indeed, kinesiophobia is relevant to shoulder pain and disability and has been found to explain around 20% of the variance in shoulder pain and disability scores [15]. While interventions such as shoulder surgery have been found to decrease kinesiophobia, their treatment outcomes are also influenced by preoperative kinesiophobia, with higher levels of preoperative kinesiophobia being associated with higher postoperative pain [16]. A comprehensive treatment program should therefore aim to identify and address these contributing factors. A common therapeutic approach is to try to break the classical conditioned-pain response (where non-harmful movements are experienced as painful after repeatedly having been paired with concurrent nociceptive stimulation) by gradual and repetitive exposure to the threatening or fearful stimulus in the relative absence of pain. Arguably, a potentially effective way to implement this is by providing false or illusory visual feedback about the currently executed bodily movement in order to decouple visual information from proprioception and nociception. It is well established that non-nociceptive sensory information, particularly visual information, can modulate pain intensity both experimentally [17] and clinically [18, 19], as a variety of information across various domains and sensations helps the brain to more accurately assess the threat level (and therefore, presumably, the resulting pain level) of a given situation. Mirror therapy is one cost-effective and simple way of providing such false visual feedback and has been shown to be effective at improving upper-limb function across a variety of pathologies [20]. Louw et al. [21] explored whether a single brief session of mirror therapy improved active flexion range of motion (AROM), pain, fear avoidance, and catastrophizing in patients with shoulder pain. The intervention involved the patient moving their non-painful arm repeatedly into flexion while observing its reflection in a mirror, giving the illusion that the painful arm is now moving painlessly through previously painful ranges. The authors found a significant increase in affected flexion range immediately post-session and suggest that this may facilitate a more rapid progression of rehabilitation. Mirror therapy has some disadvantages, however. Both the type and range of possible movements are rather limited in mirror therapy, while in reality the shoulder is a highly mobile joint capable of a huge variety of functional movements. Also, mirror therapy has a very mixed success—some patients seem to respond well; others, not at all, while some even worsen [22]. It has been suggested that variations in levels of embodiment of the moving limb may explain this disparity [23], or that variation in response may be related to levels of pain, catastrophizing, or fear avoidance [21].

Using immersive virtual reality (VR), it is possible to give the user a virtual body that can be experienced as one’s own [24–26], which can then be manipulated by the experimenter. This manipulation has been shown to have significant psychological and physiological implications and has potentially significant therapeutic potential. The virtual embodiment illusion requires three key components—firstly, the sense of body ownership (that the observed virtual body is the user’s body), the sense of self-location (that the user feels they are located in the virtual body), and the sense of agency (that the user feels control over the virtual body), each of which can be manipulated experimentally [27].

Therapeutic manipulation of virtual bodies may be relevant to particular types of chronic pain, especially in patients with deafferentation conditions, which often result in altered body image, for example in phantom limb pain [28]. Studies have shown that looking at an "embodied" virtual body [29], or body part [30, 31], changing the colour of a virtual arm that is co-located with the real arm [17], or changing its transparency [32], are factors that can modify the perception of painful stimuli in healthy subjects. VR also has the potential to positively impact the emotional factors in exercise by providing immersive, stimulating environments, personalized avatars, and gamification. Engaging in physical exercise can be challenging and leads to feelings of fatigue, or lack of motivation, especially if results are not immediate [33]. Incorporating emotional stimulation into the exercise experience can significantly impact intrinsic motivation, making it more enjoyable and sustainable. Thus, VR can enhance intrinsic motivation, making exercise more enjoyable, engaging, and sustainable, and enhancing any potential effect of therapy.

VR allows the performance of movements and tasks that are not possible in real life—whether because of immobilization, fear, pain or stiffness. Observing an embodied virtual body perform, such movements might therefore reduce movement-related fear and facilitate better real-life movements. Using embodiment of a virtual body, a false visual feedback of the body position can be provided, which can result in an unconscious enhancement of movement [34]. Subjects can still have agency over the body movements by pushing a pedal or switch with another part of the body, helping to maintain embodiment despite the break in co-location. However, it is unknown whether providing patients with such false visual movement feedback can change the onset of pain in other musculoskeletal conditions, and whether it works for embodied virtual bodies. The primary objective of our study was to ascertain whether the experience of movement of an embodied virtual arm, in the absence of actual physical movement, could effectively enhance the range of pain-free motion for patients suffering from shoulder pain related to movement. To this end, we evaluated the pain-free shoulder range of motion before and after a VR intervention in patients with movement-related shoulder pain, including rotator-cuff-related problems and adhesive capsulitis. In the next section, we describe in detail the virtual reality intervention.

## Methods

### Study design, setting and recruitment

This was a single-centre case series with a pre-post quasi-experimental design. Subjects were recruited from the outpatient rehabilitation department of Quironsalud Barcelona, a large private hospital. They were assessed for suitability according to the inclusion criteria by the head of the rehabilitation service who reviewed their medical history. Suitable patients were then recruited by telephone. Patients were considered for inclusion if they were aged 18 to 80 years old with a diagnosis of rotator-cuff-related shoulder pain (including rotator cuff tendinopathy, symptomatic rotator cuff tear, subacromial impingement, subacromial bursitis, or primary or secondary adhesive capsulitis), with a duration of 6 weeks or longer, and were currently undergoing physiotherapy treatment at the hospital. For diagnosis, a combination of clinical findings with imaging confirmation (for RCRSP) or using imaging to exclude other pathologies (in the case of adhesive capsulitis) was used. Patients were excluded if they had severe cognitive impairment (Mini-Mental State Examination test (MMSE < 24/30), severe visual deficit (if they were unable to read the introductory text when donning the HMD), epilepsy, an acute pain flare defined as greater than 7/10 pain over the previous 48 h, shoulder osteoarthritis or were pregnant.

### Sample size

Sample size was calculated using G*Power (v3.1.9.7, March 2020; [31]). Using the data from Louw et al. [21], which used a single session of mirror therapy on active flexion range of motion, we powered to detect a medium to large effect (Cohen’s *d*_*z*_ = 0.66, power = 0.80,  *α* = 0.05, correlation between measures 0.8) of the intervention on pain-free abduction range of motion with the rather conservative matched-pairs Wilcoxon signed-rank test, resulting in a required sample of 21 participants.

### Experimental procedures

**Fig. 1:**
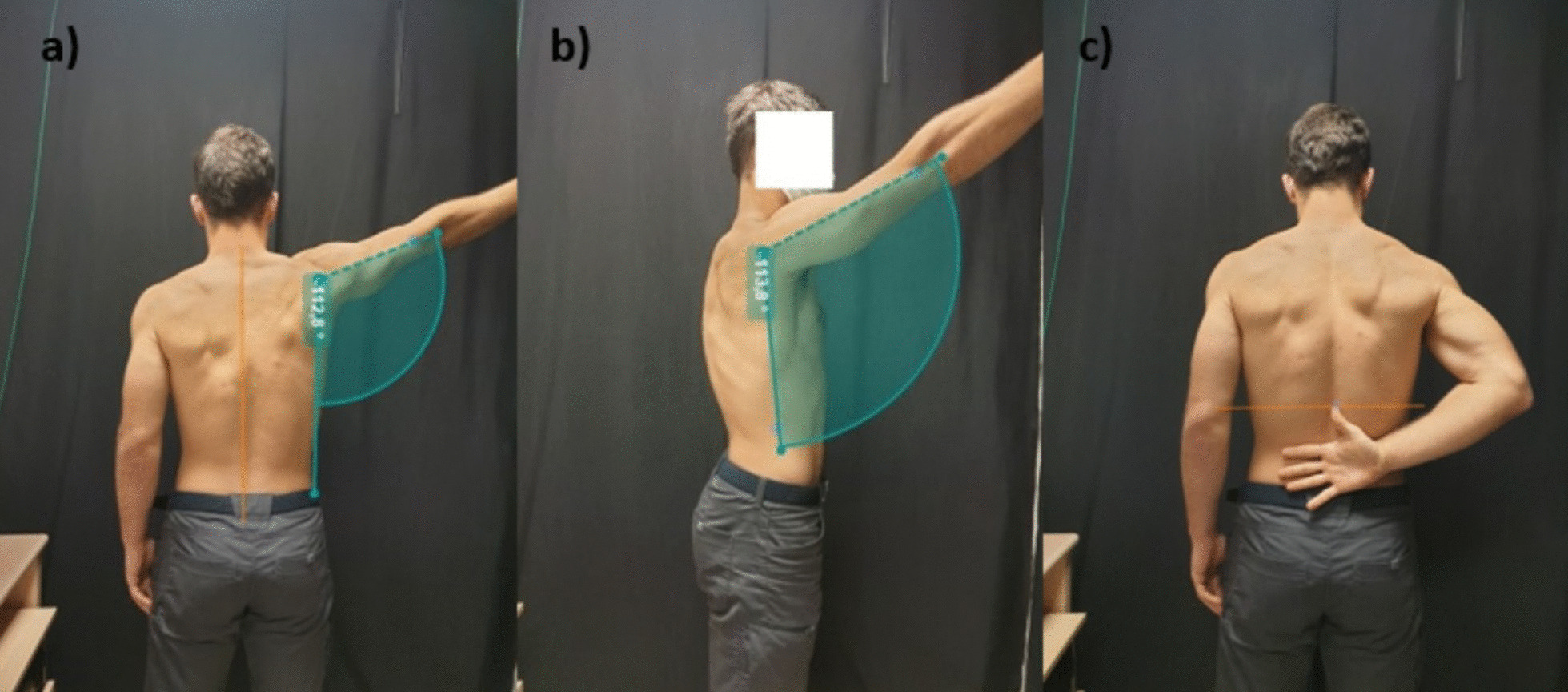
Bodily landmarks used to calculate range of motion. Shoulder (**A**) abduction, (**B**) flexion, and (**C**) hand-behind-back movements

At the start of the session, after reading and signing the consent form, the patient completed the QuickDASH and TSK-11 questionnaires (see Outcomes below). Then, their pain-free active range of shoulder abduction, flexion, and hand-behind-back was measured, as outlined above and shown in Fig. 1. Prior to performing each measurement, one of the physiotherapists would demonstrate the correct movement to the patient with clear instructions. The two physiotherapists then observed the patient perform each movement, one standing directly behind and one directly lateral to the patient. If there were any deviations from the correct plane of movement, or any compensatory head, spinal or inappropriate scapulothoracic movements (e.g., scapular elevation), the patient was informed, reinstructed on the correct movement, and the measurements repeated.

**Fig. 2:**
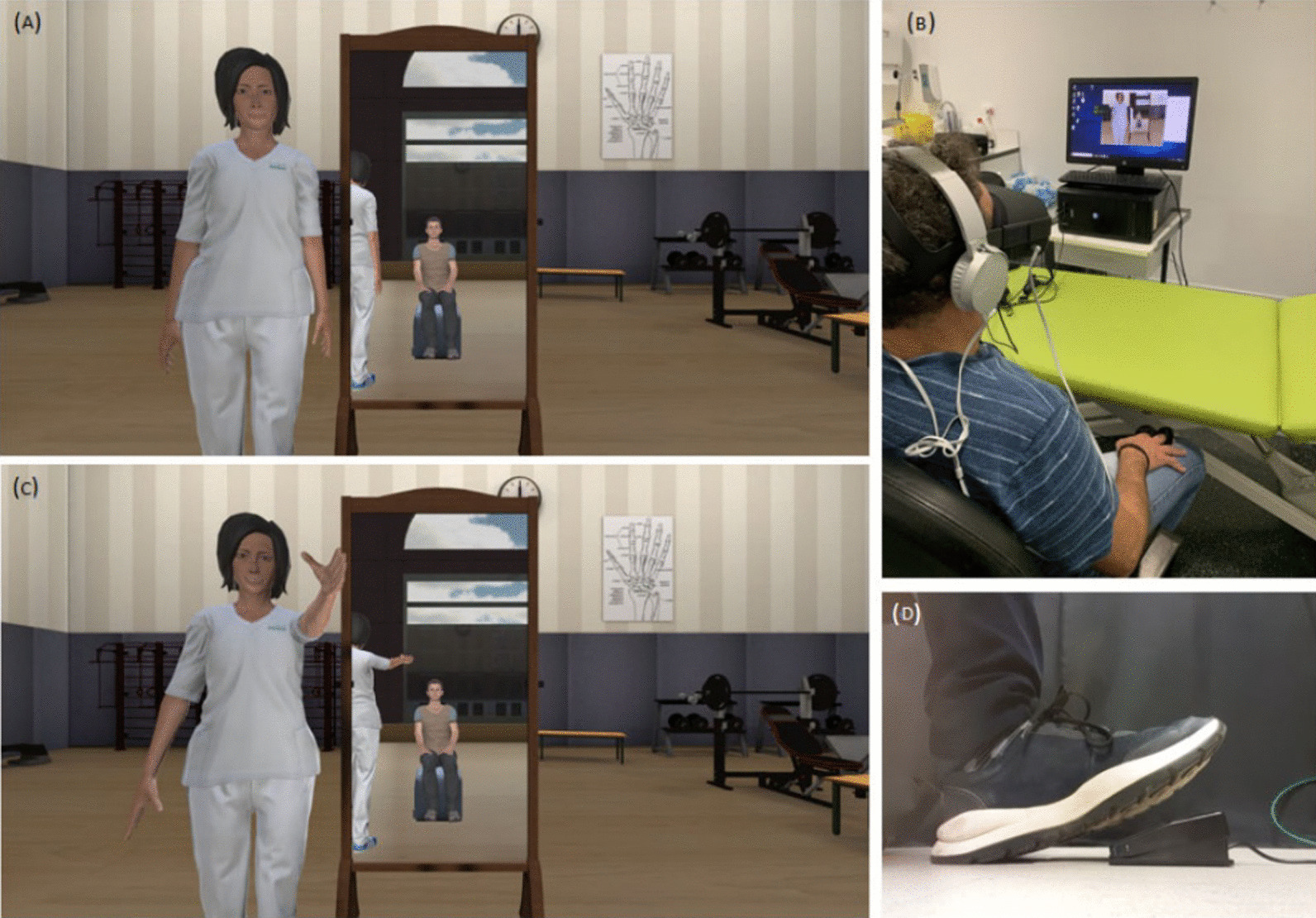
Experimental setup. **A** The virtual scene observed by the patients; note the virtual mirrors which enhance avatar-self visual feedback; **B** patient positioned quietly throughout; note the coin vibrators strapped to the fingers to provide tactile sensations during visuotactile feedback; **C** therapist demonstrating the movement/exercise to be performed; **D** pedal used to initiate the movement and confer a sense of agency over the movement

The patient was then seated with their hands resting quietly on their upper legs throughout the experiment. The patient then donned the head-mounted display (HMD) and was immersed in a virtual reality environment in which they viewed a generic gender-matched virtual body or avatar from a first-person perspective, with the upper body (head and trunk) movements of the avatar moving in synchrony with their real-life movements (Fig. 2A). Virtual mirrors to the front and side enhanced the visual feedback of the virtual body (Fig. 2B). Participants then experienced a period of immersion where they were asked to visually explore the virtual environment and their virtual body, followed by a period of synchronous visuotactile stimulation in which bouncing virtual balls were seen to touch the patients’ fingers while tactile stimulation was delivered via coin vibrators (Fig. 2C). These immersion and embodiment periods were to help generate plausible place/presence and embodiment illusions, as per the procedures first described by Sanchez-Vives et al. [24] and Slater et al. [25]. Patients were then asked to observe the therapist avatar perform eight shoulder movements (shoulder abduction, flexion, hand-behind-back; but also horizontal adduction, internal rotation/forearm pronation, and a series of three exercises that involved following the movement of a floating ball with the hand). After observing the therapist perform each movement, they initiated the equivalent movement of their virtual avatar by pressing a foot pedal (Fig. 2D) but did not actively perform the movement in real life themselves. The pedal conferred a sense of agency over the observed movement, allowing the patient to become an active actor over the body in the environment rather than a mere spectator. This is thought to activate the neural network involved in motor coordination and execution of the movement [35]. Upon pressing the pedal, the avatar performed the movement exercise three times in succession.

After the exercises were completed, the HMD was removed, and shoulder ranges of motion were measured as previously. Finally, the patient completed the virtual experience questionnaire to assess their level of immersion, agency and embodiment. To assess whether there was any carryover of the intervention, or any impact on levels of fear or disability, range of motion, kinesiophobia and disability were measured again one week later.

### Equipment

We used a head-mounted display (HMD; Oculus Quest, Facebook Inc.) with a resolution of 1440 × 1600 pixels per eye and a nominal field of view of 110°, displayed at 72 Hz to show the virtual environment, which was programmed in Unity 2019.4.10f1 (Unity Technologies, San Francisco). The virtual therapist male and female body were taken from the Rocketbox library (Rocketbox Studios GmbH, Hannover). The HMD was connected to a laptop via a Link cable. The coin vibrators were configured with an Arduino box and connected to the PC.

### Outcomes

We measured pain-free range of motion in three movements of the shoulder: abduction, flexion, and a hand-behind-back movement (mainly combined internal rotation and extension). These movements were selected as they are functional and frequently reported as painful in shoulder conditions [2, 3]. The movements were captured using frontal plane and sagittal plane video and subsequently analysed using Kinovea video analysis software (v0.9.5, Kinovea, https://www.kinovea.org), which enables half-automated movement annotation and has been demonstrated to thus facilitate very high inter- and intra-rater reliability in the measurement of shoulder range of motion [36]. Angular measurements were recorded for each movement for flexion and abduction, and patients repeated each movement. To measure the angle of shoulder abduction, lines were drawn parallel to the thoracic spine and along the midshaft of the humerus. The angle of intersection of the two lines was measured in degrees (Fig. 1A). To measure the angle of shoulder flexion, lines were drawn along the mid-axillary line and along the midshaft of the humerus. The angle of intersection of the two lines was measured in degrees (Fig. 1B). For hand-behind the back movement, the spinal level reached with the thumb was recorded, using anatomical landmarks (see Fig. 1C, and Fig. 4 caption for details of landmarks). Analyses were conducted independently by two physiotherapists. Measurements were taken at baseline and immediately post-intervention. To assess whetherr there was any carryover effect, we also took these same measurements at 1 week after the experiment; however, this was not the primary aim of the study.

We measured kinesiophobia (fear of movement) with the abbreviated version of Tampa scale of kinesiophobia (TSK-11), a patient-reported questionnaire consisting of 11 questions (scored 11–44) on movement-related fear, with higher scores indicating greater levels of kinesiophobia. The TSK-11 has been shown to be valid and reliable for shoulder pain [37], and a Spanish version has also been validated [38]. Measurements were taken at baseline and 1 week after the intervention.

We measured self-reported levels of upper-limb disability using the QuickDASH questionnaire, a patient-reported questionnaire consisting of 11 questions (with score calculated as a percentage) on symptoms and ability to do daily activities, with higher levels indicating greater disability. The QuickDash has been validated for shoulder conditions (and has also been validated in Spanish [39]). Measurements were taken at baseline and 1 week after the intervention.

Following the VR intervention, the patient completed a 7-item questionnaire regarding their virtual experience and indicated subjective levels of presence, virtual body ownership and agency over the virtual body movements (see Fig. S1; adapted from [26]). The patient had to indicate their level of agreement with a series of statements on a 7-point Likert scale (− 3 to + 3). We measured three aspects of virtual embodiment: body ownership—how much the subject feels as if the virtual body is their real body [40], agency—how much control they feel over the movement of the virtual body [41, 42], and presence—how much they feel really present in the virtual world, and that the events happening are real [43].

### Statistical analysis

All statistics were performed using Origin (v2022, OriginLab Corporation). Data were assessed for normality using visual inspection and Shapiro–Wilk testing. If the normality assumption was met, parametric testing was used (Student’s *t*-test). If the normality assumption was not met (*p* < 0.05), nonparametric testing was used (Wilcoxon signed-rank test). Additionally, nonparametric analysis was used for hand-behind-back range of motion, which used ordinal (spinal level reached) rather than interval data. Statistically significant findings for parametric data (Student’s *t*-test; *p* < 0.05) were further checked by repeating the analysis using the more conservative nonparametric Wilcoxon signed-rank test. The significance level was set at *p* < 0.05. To determine whether the VR program induced a change in pain-free range of motion, we compared pre-exposure and post-exposure range of motion by performing a paired *t*-test for flexion and abduction and a Wilcoxon signed-rank test for hand-behind-back movements (for interval and ordinal data, respectively), which is also a paired test. To determine whether aspects of the virtual reality experience, patient age, baseline levels of fear and disability and changes in range of motion were correlated, we used a Spearman’s correlation coefficient. Finally, to determine whether there was an effect of pathology/condition (adhesive capsulitis or RCRSP) or gender on degree of improvement in range of motion, we performed 3 (time) × 2 (pathology/condition or gender) repeated-measures ANOVA.

Finally, we calculated the effect size Cohen’s *d*, where *d* = mean (*c*1 − *c*2)/SD(*c*1 − *c*2) where *c*1 and *c*2 refer to pre- and post-measurements, respectively, and SD is the standard deviation of the difference scores.

## Results

### Participants and baseline assessment

Twenty-one patients (*n* = 10 males), with a mean age of 59 and mean duration of symptoms of 24 weeks, were recruited for the study and completed the VR intervention. Two patients did not return one week later to complete the follow-up measurements, one due to COVID-19 infection and the other for unknown reasons. We performed a baseline assessment consisting of a range-of-motion assessment and the QuickDASH and TSK-11 questionnaires. Full baseline participant demographics are provided in Table 1.

**Table 1:** Baseline demographic characteristics

Age (years)	59 ± 7.2
Gender	*n* = 10 males/*n* = 11 females
Condition	*n* = 6 capsulitis, *n* = 15 RCRSP
Length of symptoms (weeks)	24 ± 18.9
Kinesiophobia (TSK-11)	29.1 ± 6.1
Disability (QuickDASH)	43.8 ± 14.6

SD, standard deviation; RCRSP, rotator-cuff-related shoulder pain, QuickDASH, abbreviated version of the Disability of Arm, Shoulder and Hand questionnaire; TSK-11, abbreviated version of the Tampa scale of kinesiophobia

### Post-VR range-of-motion assessment

**Fig. 3:**
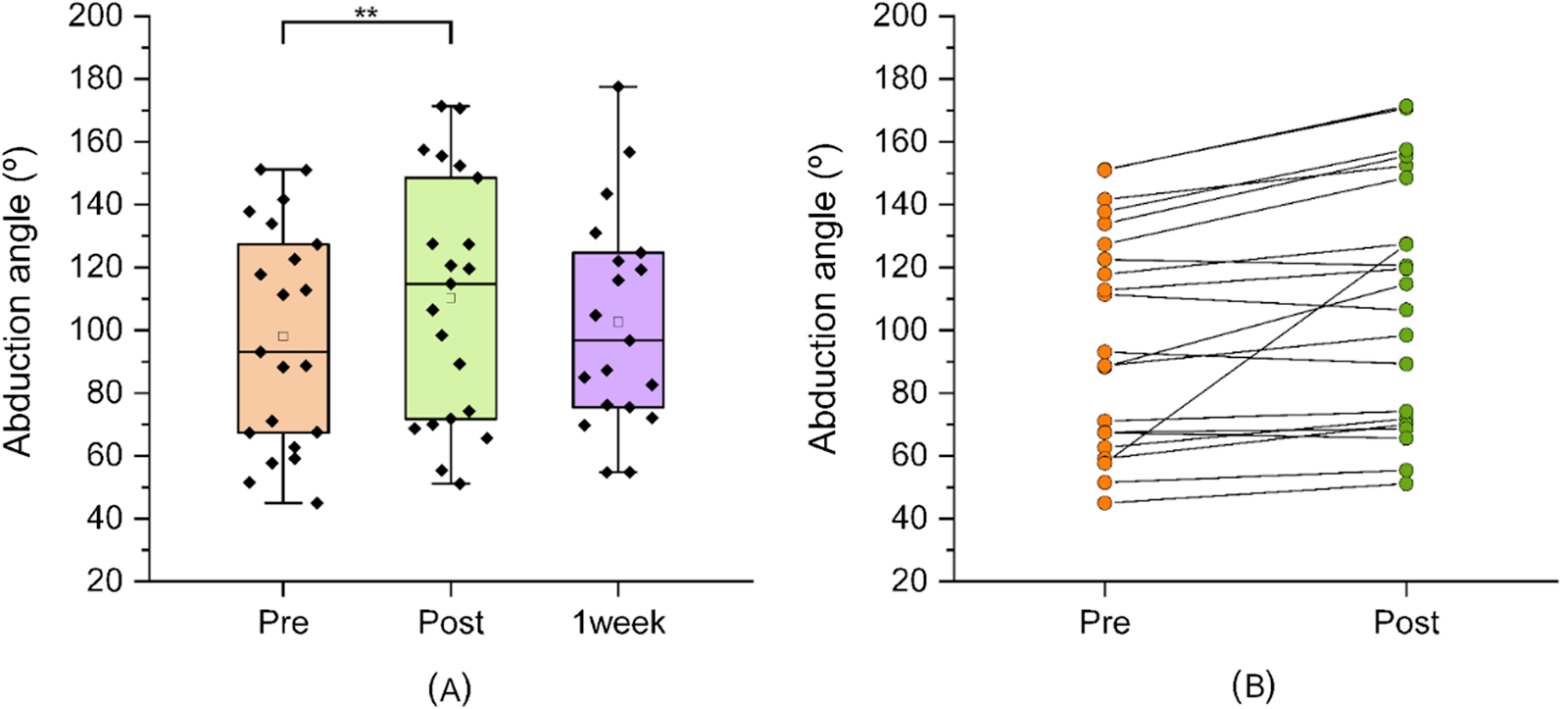
Active abduction range of motion. Boxplot showing. **A** shoulder abduction range of motion before and after the immersive VR session and 1 week later; and **B** individual patient response pre- and post-intervention. **p < 0.01

After completing the 15-min VR embodiment intervention, we re-measured each participant’s active range of pain-free shoulder range of motion. We found a significant difference in active abduction range of the affected shoulder after the intervention (mean = 110.3°; SE_mean_ = 8.58) compared with before the intervention (mean = 98.1°; SE_mean_ = 7.69). The mean improvement was 12.3° (95%CI 4.94 − 19.57; Student’s *t*-test, *P* = 0.002, Cohen’s *d* = 0.76) (Fig. 3A), which exceeded the minimum clinically important difference (MCID) for a variety of shoulder conditions reported in the literature of 2 − 10° [44]. Figure 3B shows the individual change from baseline to post-intervention.

**Fig. 4:**
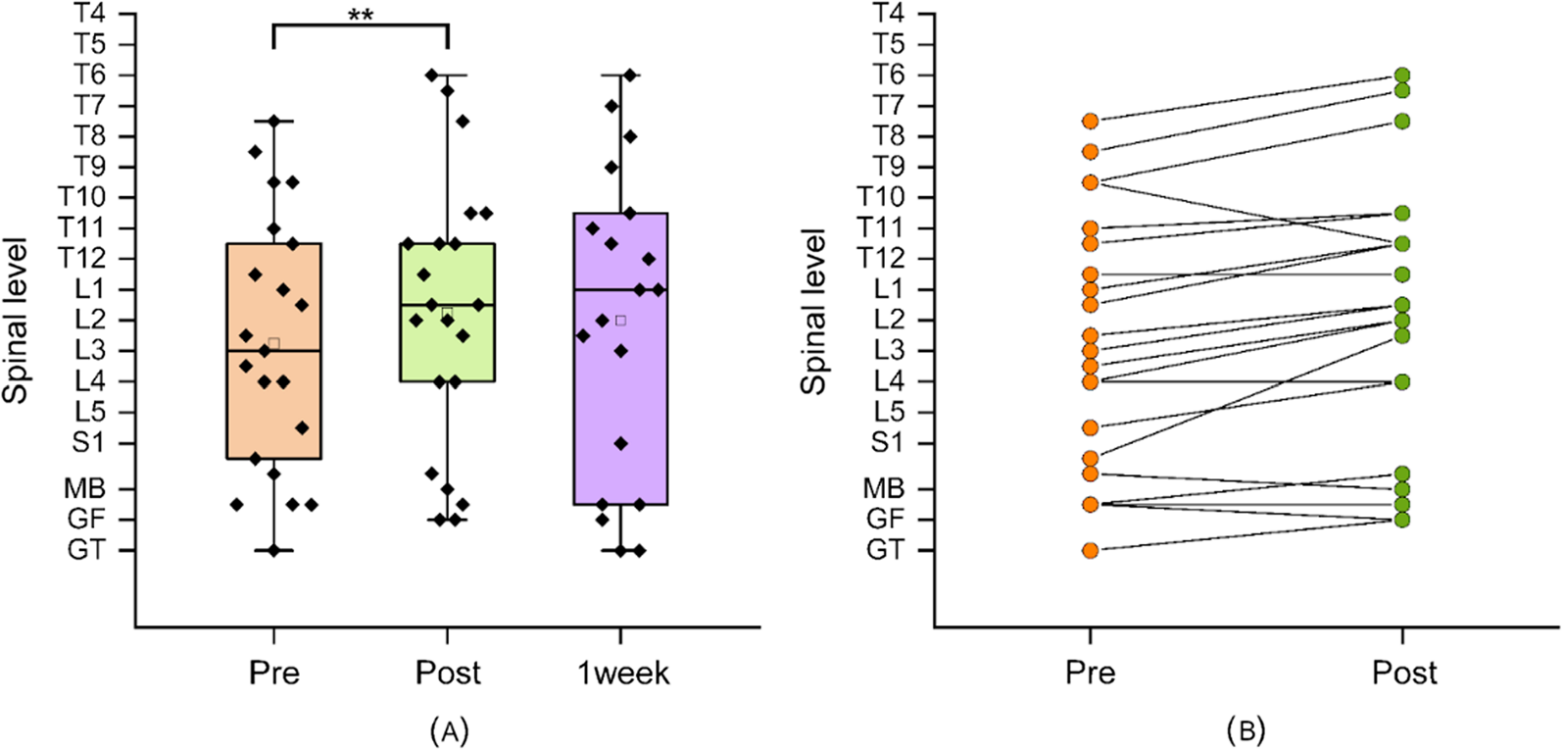
Active hand-behind-back movement. **A** Boxplot showing hand-behind-back range of motion before and after the immersive VR session and 1 week later and **B** individual response; Tx, thoracic spinal level; Lx, lumbar spinal level x; S, sacral spinal level: SIJ, sacroiliac joint; Sac, sacrum, MB mid-buttock; GF, gluteal fold; GT, greater trochanter. **p < 0.01

We also found a significant difference in active hand-behind-the-back range of motion after the intervention (median = L1/L2 junction; IQR T11/12 to L4) compared with before the intervention (median = L3; IQR T11/12 to posterior superior iliac spine, PSIS) (Fig. 4A). The median improvement was two spinal levels (95%CI 0.473  –0.916; Wilcoxon signed-rank test, *P* = 0.004; rank biserial correlation 0.778) (Fig. 4A). Figure 4B shows the individual change from baseline to post-intervention.

**Fig. 5:**
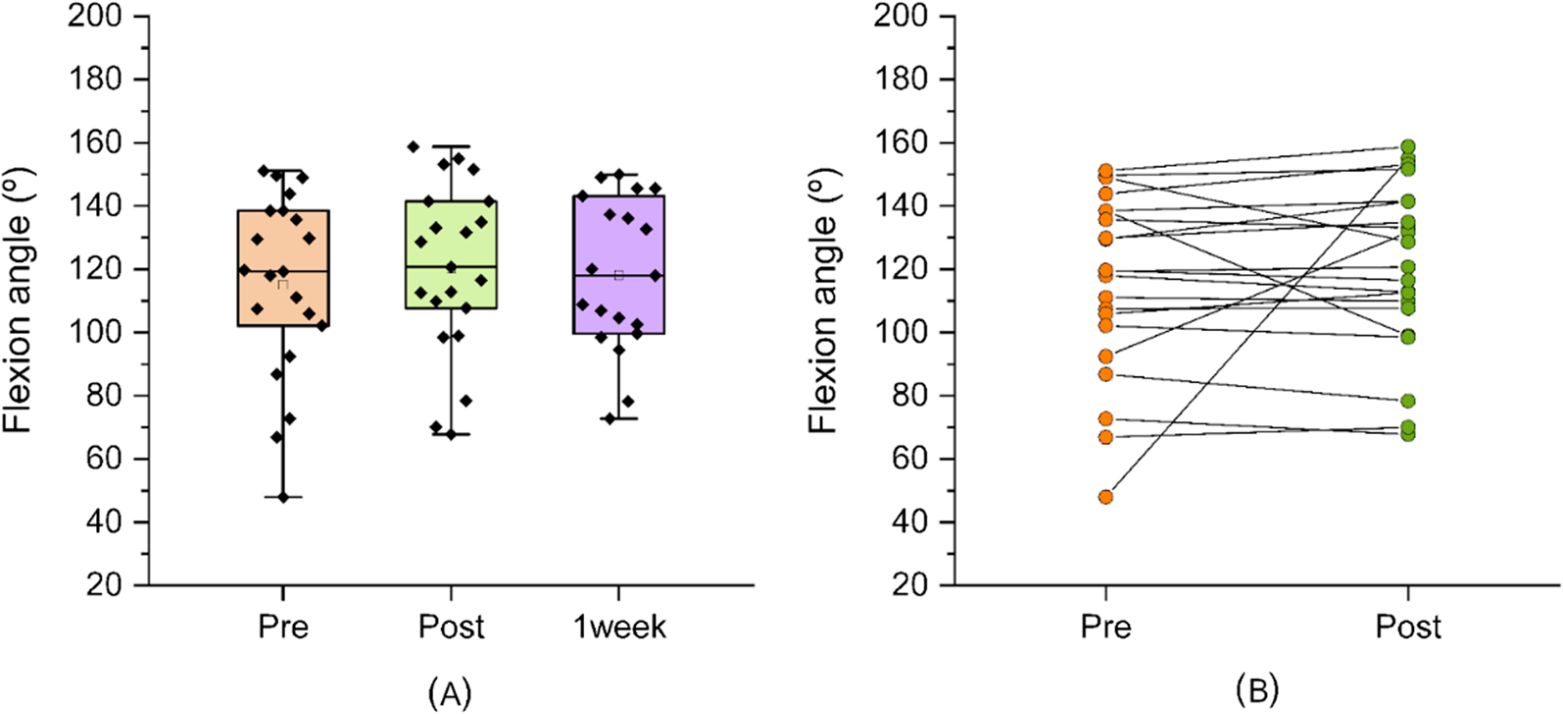
Active flexion range of motion. **A** Boxplot showing shoulder flexion range of motion before and after the immersive VR session and 1 week later and **B** individual patient response pre- and post-intervention

The active flexion range of the affected shoulder improved between baseline (mean 115.0°; SE_mean_ = 6.31) and post-intervention (mean 120.2°; SE_mean_ = 5.89); however, this was not significant (95%CI =  − 7.3 to 17.6; Student’s *t* test, *P* = 0.398, Cohen’s *d* = 0.188) (Fig. 5A). Figure 5B shows the individual change from baseline to post-intervention.

### Virtual reality experience

After reassessing the range of motion, patients completed the virtual reality questionnaire that measured aspects of the virtual embodiment experience, specifically the levels of presence in the virtual environment, virtual body ownership and agency over the virtual body’s movements.

**Fig. 6:**
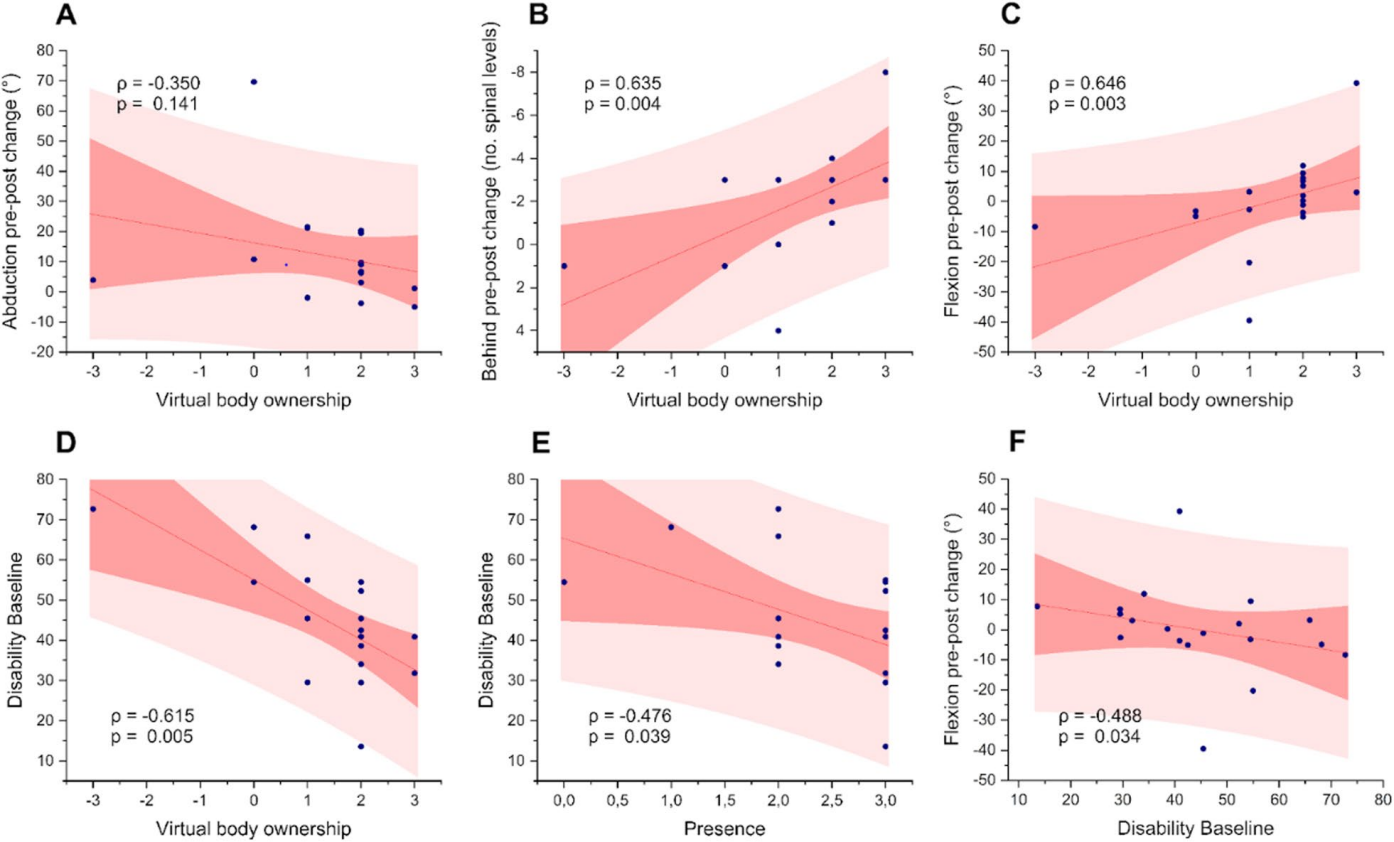
Association between outcomes: **A** Virtual body ownership vs abduction change. **B** Virtual body ownership vs hand-behind-back change. **C** Virtual body ownership vs flexion change. **D** Virtual body ownership vs baseline disability. **E** Presence vs baseline disability. **F** Disability versus flexion change

We found positive correlations between virtual body ownership and levels of improvement in both hand-behind-back movements (Spearman’s ρ = 0.635, *p* = 0.004) and flexion movements (Spearman’s ρ = 0.646, *p* = 0.003) (Fig. 6B, C). However, there was no significant correlation between virtual body ownership and level of improvement in abduction (Spearman’s ρ =  − 0.350, *p* = 0.141) (Fig. 6A).

In addition, we found that baseline levels of disability negatively correlated with levels of virtual body ownership (Spearman’s *ρ* =  − 0.615, *p* = 0.005) and presence (Spearman’s *ρ*  =  − 0.476, *p* = 0.039) (Fig. 6D, E); that is, patients who were more disabled at baseline were less likely to feel ownership over the virtual body and less likely to feel as if they were present in the virtual scene, so likely a reduced overall sense of embodiment. This may have implications in that those with higher levels of disability may be less susceptible to the embodiment illusion and therefore may need longer or more intensive exposure to multisensory stimulation if the treatment is to be effective.

There was a small negative correlation between baseline levels of disability and degree of improvement in flexion (Spearman’s *ρ*=  − 0.488, *p* = 0.034); that is, patients who were less disabled at baseline were more likely to have improved flexion movements (Fig. 6F). There was no other correlation between baseline levels fear or disability and degree of range-of-motion change. Additionally, there was no effect of pathology/condition, age or gender on degree of improvement in range of motion.

### One-week follow-up

One week after completing the VR embodiment program and assessment, the patients returned, and range of motion was reassessed. We found that the increases in range of abduction or flexion range of motion seen immediately post-treatment were not maintained at 1 week (both *p* > 0.05, Student *t*-test). For hand-behind-back movements, the median range of motion was maintained at 1 week, but this was not statistically significant (*p* = 0.943, Wilcoxon signed-rank test). Patients then completed the TSK-11 and QuickDASH questionnaires. There were no differences in levels of kinesiophobia (TSK-11) between baseline (mean = 29.1, SD = 6.1) and 1-week post-intervention (median 28.1; SD = 5.8). Additionally, there were no differences in levels of disability (QuickDASH score) between baseline (mean = 43.8, SD = 14.6) and 1-week post-intervention (mean = 41.6, SD = 17.2).

## Discussion

The present study shows that virtual embodiment and exercising the virtual arm and shoulder resulted in an increased post-intervention pain-free abduction and hand-behind-back range of motion in patients with movement-related shoulder pain. These improvements were both statistically and clinically significant, with abduction exceeding the MCID threshold of 2–10° [44]. These results suggest that a short VR intervention in which the patient experiences and observes their embodied virtual body performing previously painful shoulder movements may facilitate greater pain-free movement immediately post-intervention. The effects of this single intervention though did not persist 1 week later. It remains to be explored whether repeated virtual interventions result in persistence of the effect. Additionally, this effect appeared to be modulated by embodiment, since higher virtual body ownership levels correlated with higher levels of improvement.

There was a small but non-significant mean improvement in active flexion range of motion. It is not immediately clear why both hand-behind-back and abduction movements improved more than flexion. It is possible that the study may have been underpowered to detect an effect on flexion. Alternatively, we can speculate that since flexion is usually less painful and less restricted than the other movements, the patients’ reporting of the point of onset of pain may have been ill defined. Interestingly, these results contrast with the findings of Louw et al. [21], who used a single session of mirror therapy in a similar patient population and found an increase in flexion immediately post-treatment. Mirror therapy has also been reported to induce embodiment. Their dosage, however, was higher, with 10 repetitions versus 3 repetitions in our experiment. Aside from the difference in methodology (mirror therapy versus VR) and dosage, we note that the patients tended to look straight ahead in the virtual mirror when observing flexion, unlike mirror therapy where they would look to the side.

Given that we found significant correlations between levels of virtual body ownership and degree of improvement in two of the three measured movements, we can suggest that embodiment may play a significant role in the measured improvement.

Few other studies have examined the use of providing illusory visual feedback using immersive VR in clinical populations [45]. Harvie et al. [19] demonstrated that pain onset is altered in motion-induced chronic neck pain when false visual feedback is provided through a head-mounted display (HMD) using immersive VR—when less movement is observed, patients moved their head further before reporting pain onset, and vice versa. However, the patients were not embodied in a virtual body for this study. More recently, Matamala-Gomez et al. [35] showed that frequent use of an embodiment-based immersive VR training program improved functional motor ability of the upper limb in patients immobilized following a distal radius fracture compared with non-immersive VR or standard treatment, and the results in the current study are consistent with these findings.

### Mechanisms of effect

The exact physiological mechanism for the improved range of motion is not fully understood, but there are a number of possibilities that we will now discuss.

#### Changes in muscle activation

We can speculate that our VR intervention may induce a change in muscle activation patterns, facilitating the visualized movement due to reduced muscle guarding post-intervention. Muscle guarding in patients with shoulder pain is common and could be considered as a protective mechanism or a response to underlying pathology, or a combination of the two. However, guarding has been shown to predict the development of chronic pain [46], where it becomes a maladaptive behaviour. Cognitive or emotional factors such as kinesiophobia, anxiety or catastrophizing can influence behaviour and result in limitations to movement, and addressing these factors can help improve range of motion. At least some of the movement restriction observed in adhesive capsulitis has been shown to be related to excessive muscle guarding in addition to the capsular restriction itself [47]. In this case, experiencing painful or “dangerous” movements without pain in VR might reduce this protective guarding response.

Ngomo et al. [8] have shown that individuals with rotator cuff tendinopathy have decreased corticospinal excitability of the infraspinatus muscle on the affected versus unaffected side. Although cause and effect are not clear, another plausible mechanism for the effect of the intervention is a restoration of normal rotator cuff excitability levels, resulting in a normalization of muscle activation levels and biomechanics. Traditional mirror therapy appears to have a facilitatory effect on corticomotor excitability [48, 49], but the effect of virtual embodiment on corticomotor excitability is less clear. Movements of an embodied virtual arm have been found to induce activity as recorded in the electromyogram however [25]. Transcranial magnetic stimulation combined with electromyography could be a useful way of untangling the potential effect of the intervention on corticomotor excitability in future studies.

#### Breaking the associative learning response

The second mechanism relates to a classical conditioning or associative learning response. Bodily movements usually involve a combination of motor, visual, and proprioceptive sensory information, and when these movements become repeatedly and strongly associated with nociceptive input, over time these non-nociceptive sensations may be sufficient to trigger pain with or without nociception [50]. This associative learning response may explain how pain-related fear and behaviour become ingrained over time, consistent with the fear-avoidance model of Vlaeyen and Linton [51] and with the operant learning theory of pain [52], which holds that reinforcing pain behaviours leads to their maintenance. Experiencing the illusion of a normally moving limb helps to break this association—by experiencing only the visual sensation of an owned arm movements in the relative absence of proprioceptive and motor input, we suggest this may start to separate and disentangle the associative link of pain with movement by inducing respective plastic changes in the central nervous system. Violating the expectation of pain in this way is a powerful therapeutic tool, as it induces a large prediction error, which in turn causes a significant updating of our internal models used to generate future predictions about the body in its environment, and forms a key component of therapeutic approaches to chronic pain such as exposure therapy [53].

There is also an apparent contradiction here since the visual evidence of movement by itself *was* sufficient in a few patients to induce pain. This phenomenon has also been noted in conditions with high levels of central sensitization (e.g., CRPS [54] or phantom limb pain [55]) and supports a top-down mechanism, since pain is produced in the absence of any motor or proprioceptive input from the limb. We speculate that these patients may have been sufficiently centrally sensitized that their second-order nociceptors fired spontaneously and generated a pain experience. An alternative mechanism may be related to activation of mirror neurons [56], which activation of which may be enough to generate a painful experience in the absence of input from the limb, or a more general nocebo effect, which would involve more distributed cortical processes [57].

#### Alteration of cortical representations

Structural and functional alterations in the primary somatosensory and motor cortices are associated with certain types of persistent pain, including shoulder pain [8, 9], and these changes have been proposed to be a potential driver of central sensitization [58]. Whether these cortical changes are a cause or result of persistent pain is unclear, as is whether they are a consequence of pain or of associated disuse. However, they do appear to resolve alongside pain (or function) resolution and trying to restore normal cortical representations is therefore a viable therapeutic target. By action planning and then actually experiencing illusory movements of an embodied virtual limb, we can repeatedly activate the neural networks involved in motor action. This may have an effect reducing cortical atrophy and perhaps start to reverse any cortical shrinkage of the shoulder representation in the sensory and motor cortices. It is unlikely, however, that a single short VR session would have a significant effect on structural alterations such as the loss of sulcal depth seen in chronic shoulder pain [9]. Topographical alterations, in contrast, can change much more quickly—even within minutes [59]—especially where the alterations involve masking/unmasking of previous synaptic connections. However, there is limited evidence for altered cortical representations in shoulder pain (see [60, 61] for a more general discussion), and this mechanism is therefore less likely to be predominant. Additionally, we did not monitor pain intensity directly, only pain-free range of motion; however, if central sensitization were reduced, we would reasonably expect to see improved pain-free range of motion as well as a reduction in pain intensity.

#### Other possible contributing mechanisms

It is unlikely that the overall improvement in pain-free range of motion is simply be due to a stretching effect due to movements undertaken in the initial assessment, since the 20-min VR program, in which the patient’s shoulder is completely static, is almost certainly long enough to eliminate any such potential gains. In addition, placebo analgesia, in which pain thresholds are increased simply through expecting less pain, may well play a role [57]. Patients, having experienced their limb moving normally and without pain, may simply be expecting less pain when they move in reality. Such “nonspecific” effects are still likely therapeutically useful and form a part of all “active” therapeutic treatments.

The mechanisms outlined here are not necessarily mutually exclusive, and further experimental studies with appropriate controls are required to disentangle the neurophysiological mechanisms involved with embodiment illusions (for a more detailed discussion, see [45]).

### Carryover effects

There was no significant carryover of the intervention on any of the outcomes. At 1 week, none of these improvements were maintained and neither were there any changes in kinesiophobia or disability from baseline. There are several explanations for this. We would not reasonably expect a single short session of VR to have a lasting effect on range of motion, fear or disability. Whether repeated or longer sessions are more likely to have a carryover effect is unknown. Generally, the prescribed dosage for traditional mirror therapy is much higher in terms of both the number of sessions, and the number of times a movement is observed, so we would reasonably expect some sort of cumulative effect with repeated VR sessions. Future studies should explore optimal dosage in terms of length and number of sessions in further depth.

### Outlook

The virtual embodiment and exercising approach described in this study have different potential future applications for pain and physical rehabilitation. Exercising the virtual body can have an impact at different levels, from facilitating movements to reducing kinesiophobia. The program could be combined with traditional physiotherapy, for example as part of a warm-up session that precedes the main treatment. It can also be used in patients with high levels of pain, anxiety or kinesiophobia as part of a cognitive-behavioural exposure therapy to address kinesiophobia and the resulting movement avoidance. Other approaches, such as gamification, have also been pursued for this purpose [62] and could be as eventually integrated.

### Limitations

There were several limitations to the current study. No control group was included, so we cannot fully rule out nonspecific effects such as the novelty of the intervention. In addition, while we recorded outcomes at 1-week post-intervention, the patients were all undergoing regular physiotherapy treatment at the time, so we cannot attribute any changes seen at 1 week to the intervention just to the VR intervention.

Dosage may not have been sufficient (with just three repetitions for each movement), and we may have seen greater improvements with more repetition (standard mirror therapy dosage is usually higher). However, this would have meant either spending longer in VR or reducing the number of exercises, which could reduce patient engagement. It was felt that on balance a wider variety of exercises with less repetitions were optimal. Generally, it should also be considered that longer interventions may increase the chances of inducing motion sickness, although with these contents, where the patient was static, the chances were lower.

We did not monitor levels of fear or disability immediately post-treatment since it requires time to see and experience changes with the patient experimenting and testing movements and activities; however, with just a single session, a significant effect on these factors is less plausible.

## Conclusions

A single immersive VR session in which patients with movement-related shoulder pain observed their embodied virtual body performing previously painful movements was sufficient to induce clinically and significantly improvements in abduction and hand-behind-back range of motion. No such improvement was seen for flexion. The effect on range of motion appeared to be correlated with levels of virtual body ownership and therefore embodiment. Virtual embodiment may be a useful tool to improve active range of motion in patients with movement-related pain and may help expedite rehabilitation and recovery from a variety of such conditions, especially in the early stages of rehabilitation where patients are often highly anxious or fearful and avoidant of movement.

## Data Availability

The datasets used and/or analysed during the current study are available from the corresponding author on reasonable request.
